# Supplementary material for: DNA methylation in mice is influenced by genetics as well as sex and life experience
Source: Nat Commun. 2019 Jan 18;10:305. doi: 10.1038/s41467-018-08067-z (PMC6338756; doi:10.1038/s41467-018-08067-z)
Supplement: Supplementary file 1 — Supplementary Information [file 41467_2018_8067_MOESM1_ESM.pdf]

Grimm et al., Supplementary Figure 1

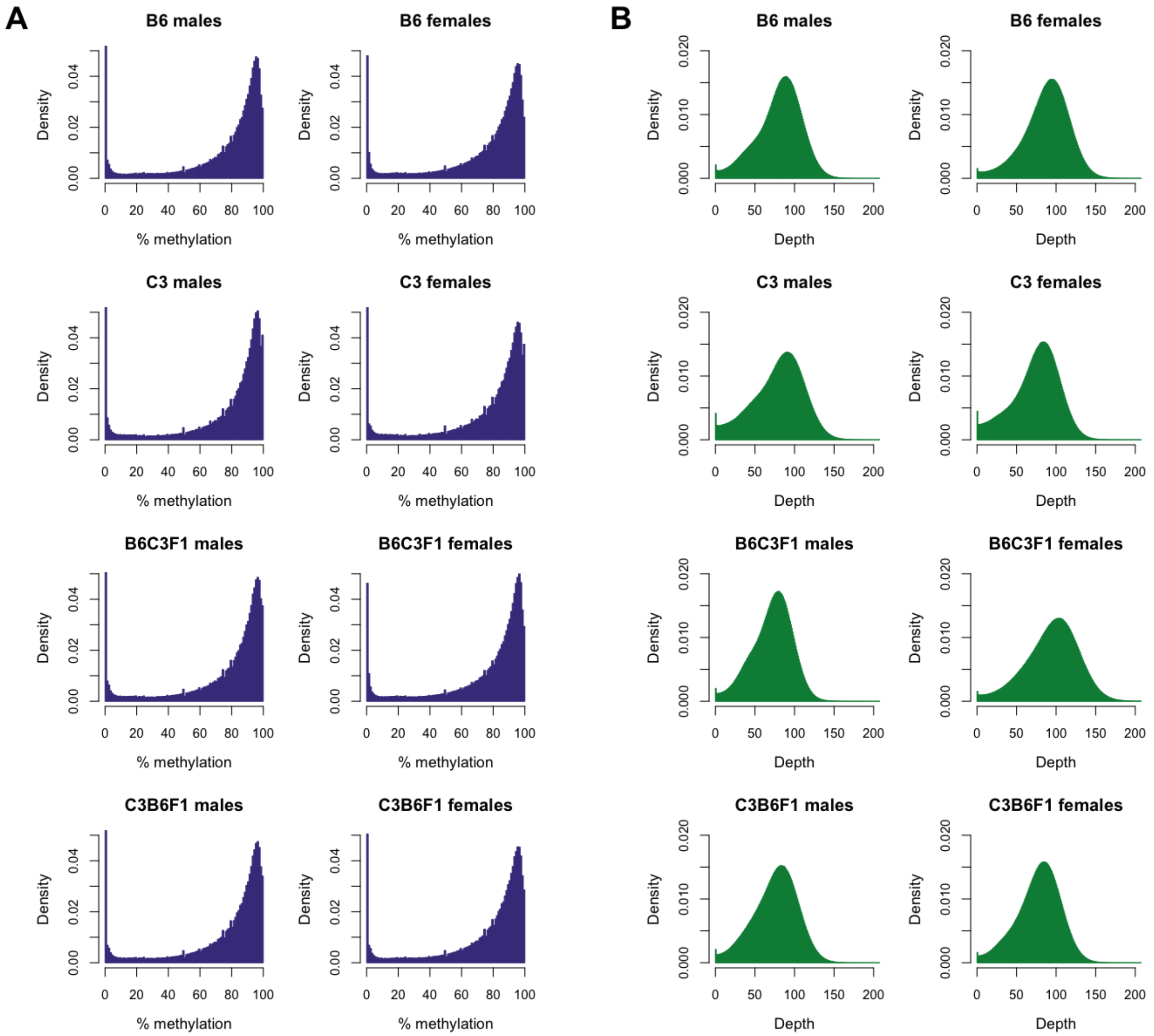

Supplementary Figure 1. Genome-wide CpG methylation and sequencing depth.

The calculated %methylation (**A**) and sequencing depth (**B**) per CpG site for merged data from 3 replicate animals per genotype. Reciprocal cytosines from opposite strands in CpG context are considered as a single site. Only sites for which the CpG context has been validated in both the B6 or C3 parental genomes are included; N=19,751,474 (excluding sites with zero depth in the %methylation panels).

Grimm et al., Supplementary Figure 2

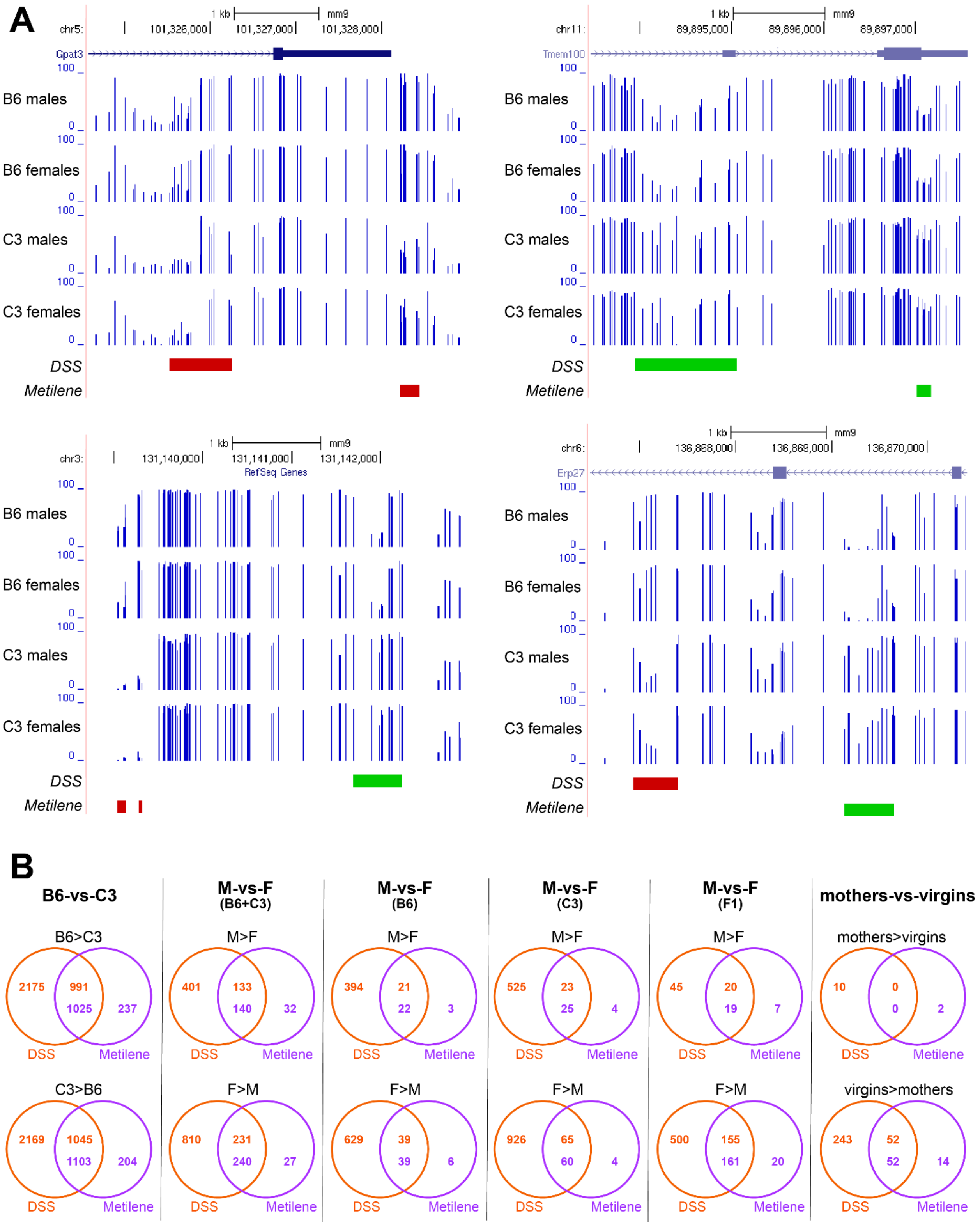

Supplementary Figure 2. Comparison of computational approaches for calling Differentially Methylated Regions.

A. Genome browser view of exemplar DMRs called by only one computational tool. DMRs are indicated by horizontal bars: B6>C3 DMRs (red) and C3>B6 DMRs. Computational tools are indicated at the bottom of each panel.

B. Concurrence of computational tools. The Venn diagrams indicate the overlapping and unique DMR calls for each comparison indicated in the figure.

Grimm et al., Supplementary Figure 3

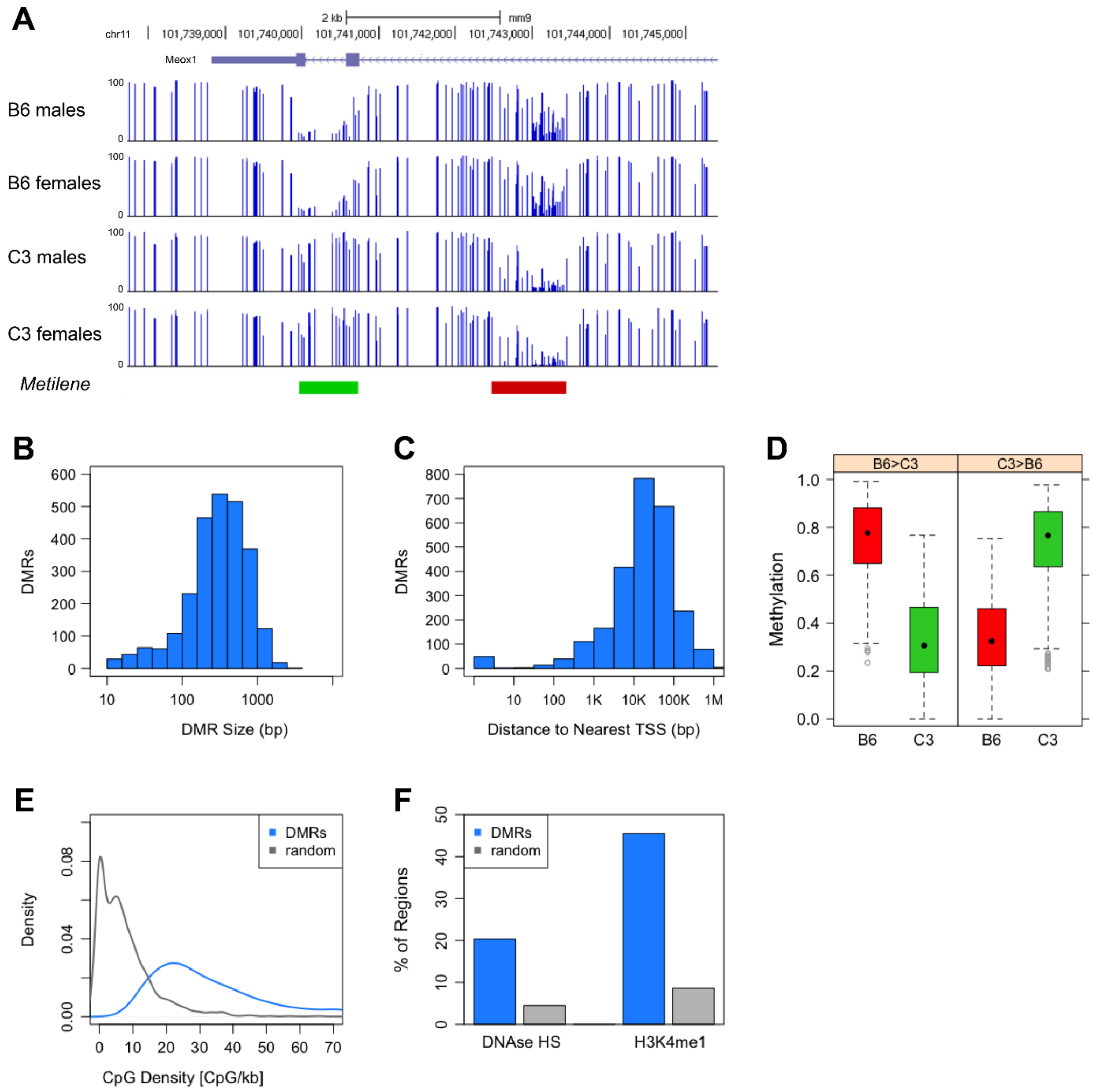

Supplementary Figure 3. DMRs between B6 and C3 parental animals, identified by Metilene.

A. Genome browser view of example B6>C3 DMR (red) and C3>B6 DMR.

B. Distribution of DMR size, N=2569.

C. Distribution of distance between DMR and nearest TSS (based on RefSeq gene models as of Feb 8 2016), N=2569.

D. Distribution of methylation level in Metilene DMRs, by strain and DMR polarity. N=1262 B6>C3 DMRs and 2570 C3>B6 DMRs. The box depicts the 25th to 75th percentiles (color by genotype where B6 is red and C3 is green), the black dot is the median, the whiskers extend to data points up to 1.5*IQR beyond the box, and open gray circles are data points outside the whisker range.

E. Distribution of CpG density at Metilene DMRs (N=2569), compared to size-matched random genomic regions (N=2569).

F. Overlap of Metilene DMRs (N=2569) with DNase hypersensitive sites (UCSC Genome Browser) and H3K4me1 enriched regions (ENCODE peaks ENCFF001XXZ), compared to the average overlap observed for 1000 iterations of size-matched random genomic regions.

Grimm et al., Supplementary Figure 4

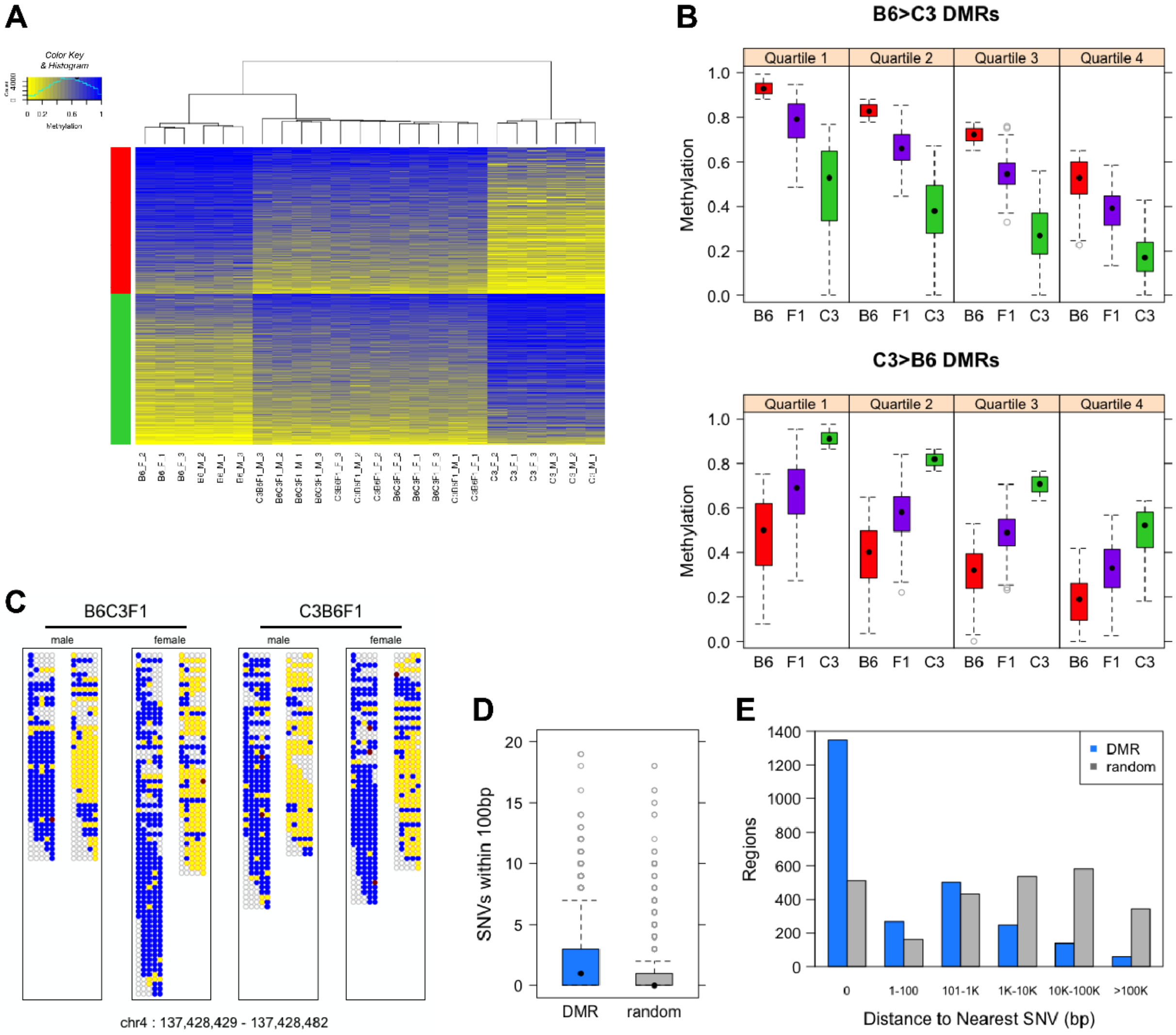

Supplementary Figure 4. DNA methylation patterns at Metilene genotype-DMRs are recapitulated on parental alleles in F1 progeny.

A. Heatmap view of weighted methylation scores per B6-vs-C3 Metilene DMR (N=2569) per animal. Hierarchical clustering of animals performed by R package ‘amap’ (hcluster with method=euclidean and link=average). DMRs were split by direction (B6>C3: red bar, or C3>B6: green bar) then sorted by average methylation score over all 24 animals.

B. Distribution of average weighted methylation scores, with DMRs (N=2569) split into quartiles according to decreasing methylation score of the hypermethylated parental genome.

C. Methylation inheritance in F1 progeny at the read level for an exemplar Metilene DMR, organized as described for Fig3C.

D. Number of SNVs local to DMRs (N=2569), compared to size-matched random genomic regions (N=2569).

E. Distribution of distance to the nearest SNV for DMRs (N=2569) or size-matched random genomic regions (N=2569). Distance=0 indicates that at least one SNV is found within a region of a given type. In the box-and-whisker plots, the box depicts the 25th to 75th percentiles, the black dot is the median, the whiskers extend to data points up to 1.5*IQR beyond the box, and open gray circles are data points outside the whisker range.

Grimm et al., Supplementary Figure 5

A B6>C3 DMRs

Motif	*Name*	*pvalue*
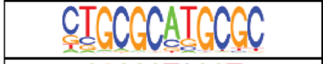	NRF1(NRF)	1e-9
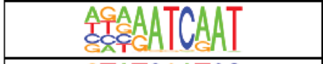	Cux2(Homeobox)	1e-9
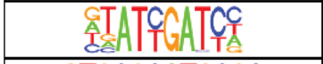	HNF6(Homeobox)	1e-8
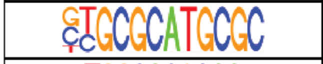	NRF(NRF)	1e-8
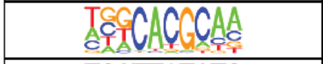	Arnt:Ahr(bHLH)	1e-8
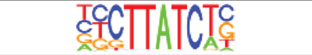	Gata2(Zf)	1e-6
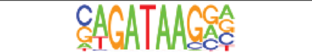	Gata1(Zf)	1e-5
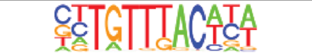	Foxa2(Forkhead)	1e-4
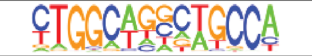	Tlx?(NR)	1e-4
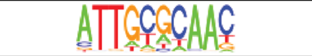	CEBP(bZIP)	1e-4
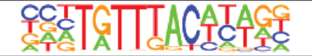	Foxa3(Forkhead)	1e-4
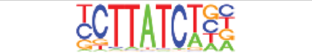	Gata6(Zf)	1e-4
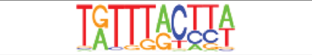	FOXM1(Forkhead)	1e-4
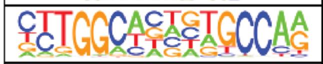	NF1(CTF)	1e-4
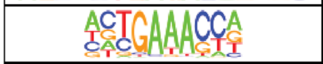	IRF4(IRF)	1e-3
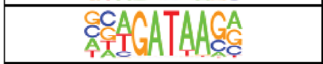	Gata4(Zf)	1e-3
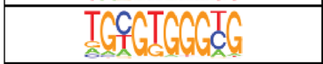	Egr1(Zf)	1e-3
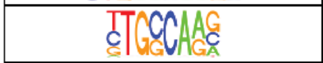	NF1-halfsite(CTF)	1e-3
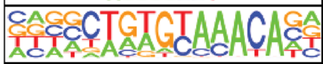	Fox:Ebox (Forkhead,bHLH)	1e-3
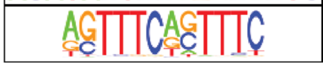	ISRE(IRF)	1e-3

B C3>B6 DMRs

Motif	*Name*	*pvalue*
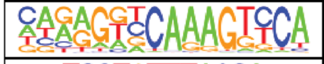	HNF4a(NR),DR1	1e-10
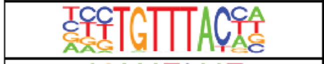	FOXP1(Forkhead)	1e-8
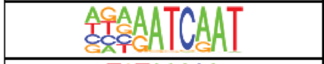	Cux2(Homeobox)	1e-8
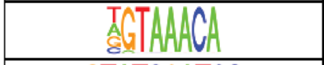	Foxo3(Forkhead)	1e-7
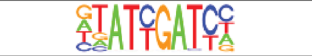	HNF6(Homeobox)	1e-7
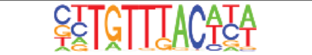	Foxa2(Forkhead)	1e-6
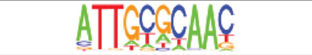	CEBP(bZIP)	1e-6
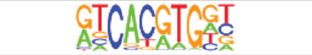	Usf2(bHLH)	1e-6
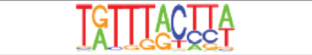	FOXM1(Forkhead)	1e-5
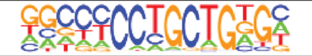	Zic3(Zf)	1e-5
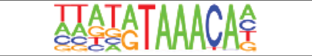	Foxf1(Forkhead)	1e-4
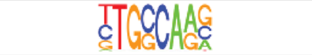	NF1-halfsite(CTF)	1e-4
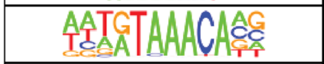	FoxL2(Forkhead)	1e-4
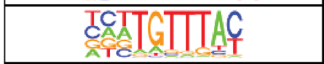	FOXK1(Forkhead)	1e-4
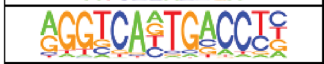	FXR(NR),IR1	1e-4
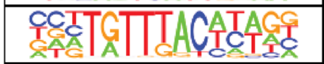	Foxa3(Forkhead)	1e-4
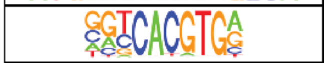	USF1(bHLH)	1e-3
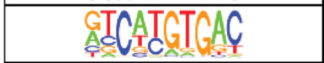	MITF(bHLH)	1e-3
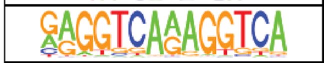	TR4(NR),DR1	1e-3
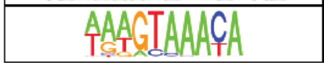	FOXA1(Forkhead)	1e-3

Supplementary Figure 5. Enriched motifs in Metilene genotype-DMRs.

A. Top 20 enriched TF motifs in B6>C3 Metilene DMRs (after expanding DMR size to a minimum of 401 bp) according to HOMER v4.9.1

B. Top 20 enriched TF motifs in C3>B6 Metilene DMRs (after expanding DMR size to a minimum of 401 bp) according to HOMER v4.9.1

Grimm et al., Supplementary Figure 6

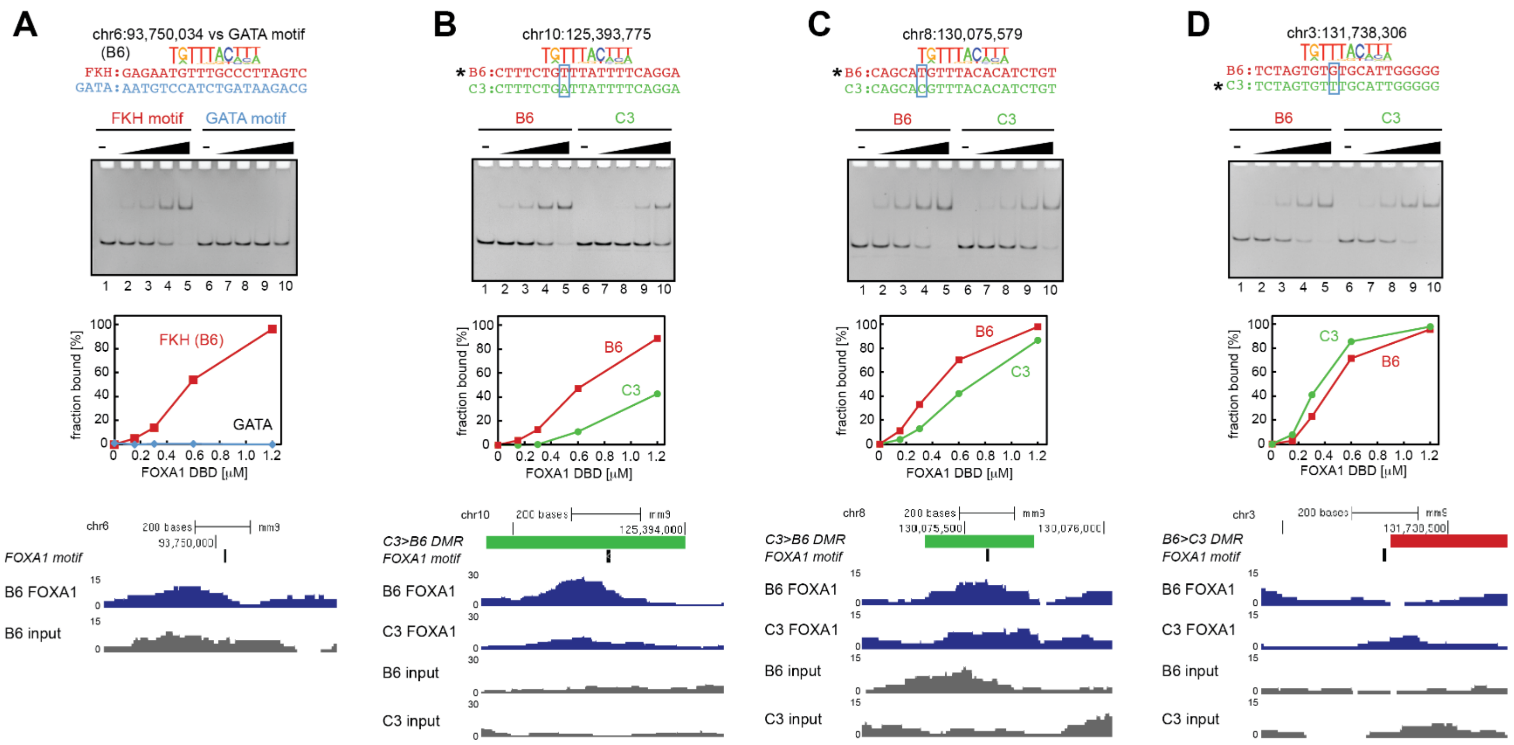

Supplementary Figure 6. FoxA1 binding at DMRs is impacted by local DNA sequence.

A-D. DNA-binding analyses of FOXA1 DBD with SNV-containing DNAs. The genomic positions and DNA substrates are indicated at the top of each panel with a representative FOXA1 motif. The position of the SNV is highlighted with light blue boxes. The band intensities were calculated using ImageJ software. 20 bp double-stranded DNAs were incubated with the FOXA1 DBD. The concentration of FOXA1 DBD was as follows: 0 μM, lanes 1,6; 0.15 μM lanes 2,7; 0.3 μM, lanes 3,8; 0.6 μM, lanes 4,9; 1.2 μM, lanes 5,10. The protein-DNA complex was separated by native-polyacrylamide gel electrophoresis. Browser tracks of ChIP-seq data for each locus is shown below the DNA binding data.

Grimm et al., Supplementary Figure 7

A. **B6 Dam** **C3 Dam**

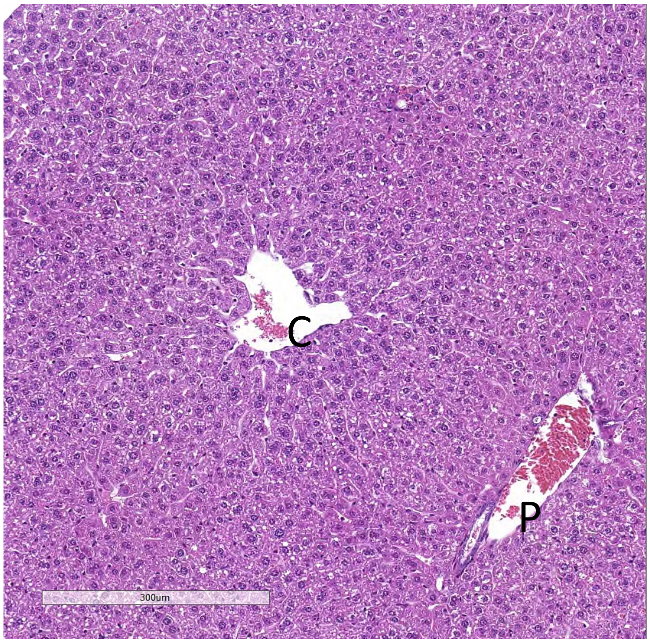

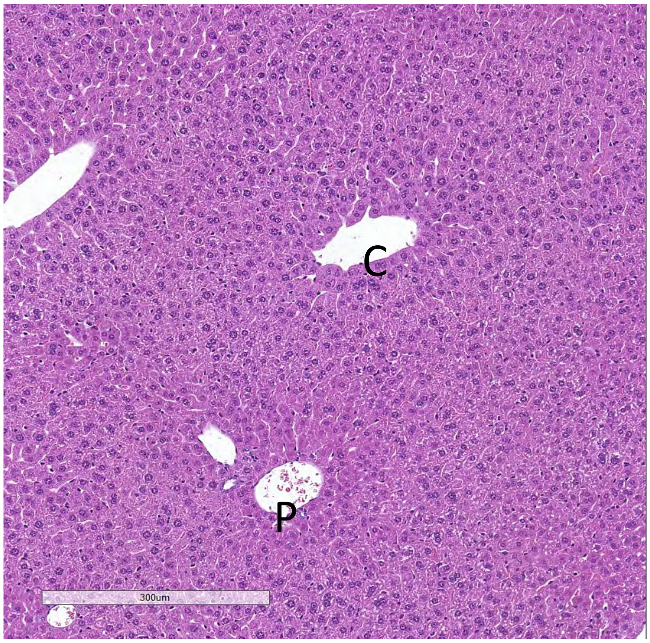

B. **B6C3F1 virgin female** **C3B6F1 virgin female**

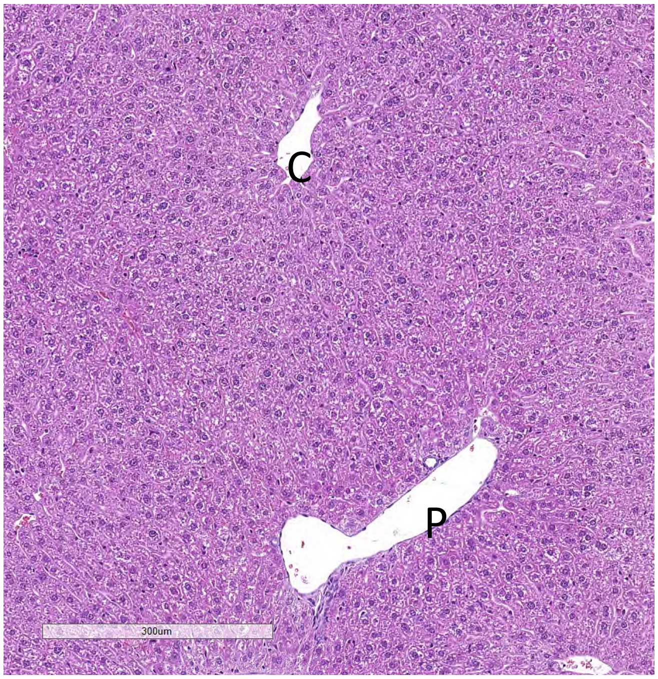

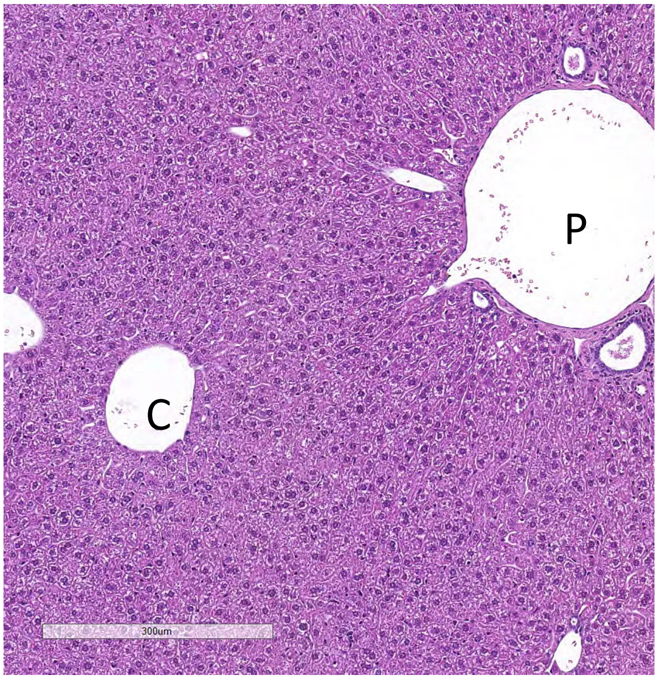

C.

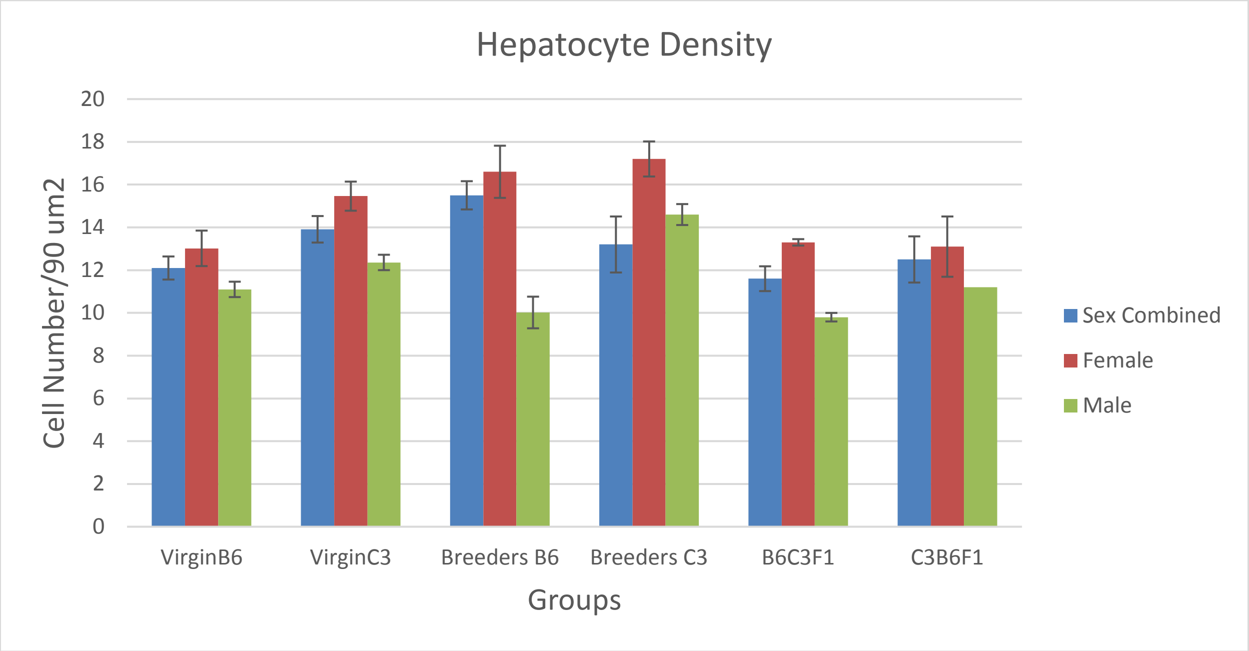

Supplementary Figure 7. Histologic analysis of study animal livers

A-B. A single H&E-stained liver section was evaluated for each animal to assess any histopathologic changes with attention to possible hypertrophic changes. There was no evidence of hypertrophy in any of the female mice from either the B6 or C3 strains in any of the animal groups. Some animals exhibited glycolysis secondary to *ad libitum* distribution of chow as evidenced by the heterogeneous zonal distribution of hepatocytes with vacuolated cytoplasm. The degree of glycolysis varied between and within groups. No other significant lesions were noted in the liver of any female mice. A representative image of an H&E stained, digitally scanned section from a female mouse of each strain and within each group was captured at 10X. Panel A depicts liver sections from B6 and C3 dams. Panel B depicts sections from B6C3F1 and C3B6F1 as noted in the figure.

C – central vein; P – periportal vein

C. Hepatocyte counts. Female and Male mice from three groups of animals: virgins (B6 and C3), breeders (B6 dams and C3 dams) and offspring (B6C3F1 and C3B6F1), were evaluated for hypertrophy.

Hypertrophy assessment was based on hepatocyte density counts generated from the same, H&E-stained liver sections scanned on the Aperio ScanScope XT instrument (Vista, CA) using ImageScope software, (v11.2.0.780, Aperio). Hepatocyte nuclei from ten, non-overlapping, 90x90 μm^2 fields were analyzed per section to determine cell density.

Grimm et al., Supplementary Figure 8

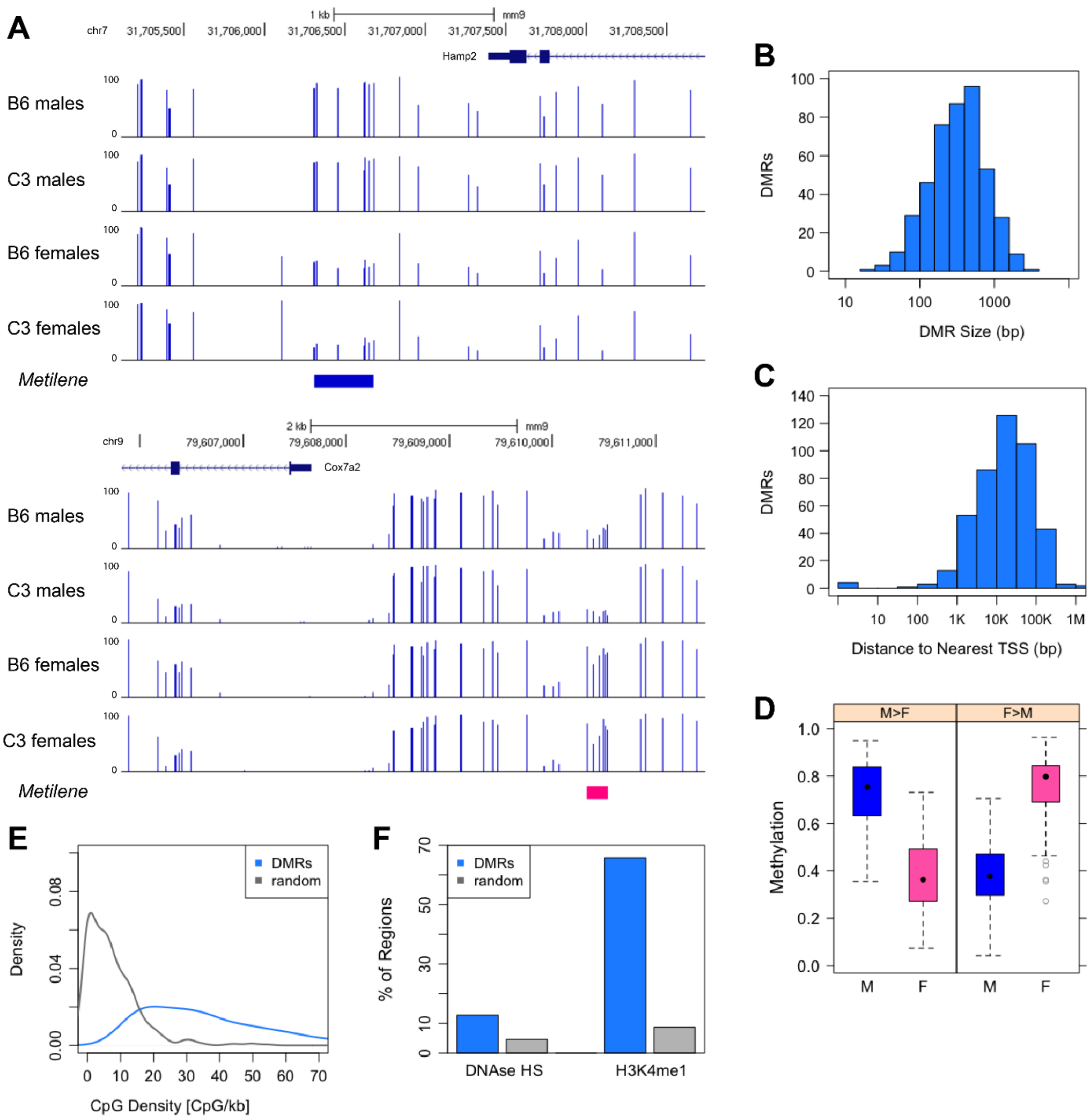

Supplementary Figure 8. DMRs between Male and Female parental animals, identified by Metilene.

A. Genome browser view of example M>F DMR (blue, top) and F>M DMR (pink, bottom).

B. Distribution of DMR size, N=439.

C. Distribution of distance between DMR and nearest TSS (based on RefSeq gene models as of Feb 8 2016), N=439.

D. Distribution of methylation level in Metilene DMRs, by strain and DMR polarity. N=267 F>M DMRs and 172 M>F DMRs. The box depicts the 25th to 75th percentiles (color by sex where male is blue and female is pink), the black dot is the median, the whiskers extend to data points up to 1.5*IQR beyond the box, and open gray circles are data points outside the whisker range.

E. Distribution of CpG density at Metilene DMRs (N=439), compared to size-matched random genomic regions (N=439).

F. Overlap of Metilene DMRs (N=439) with DNase hypersensitive sites (UCSC Genome Browser) and H3K4me1 enriched regions (ENCODE peaks ENCFF001XXZ), compared to the average overlap observed for 1000 iterations of size-matched random genomic regions.

Grimm et al., Supplementary Figure 9

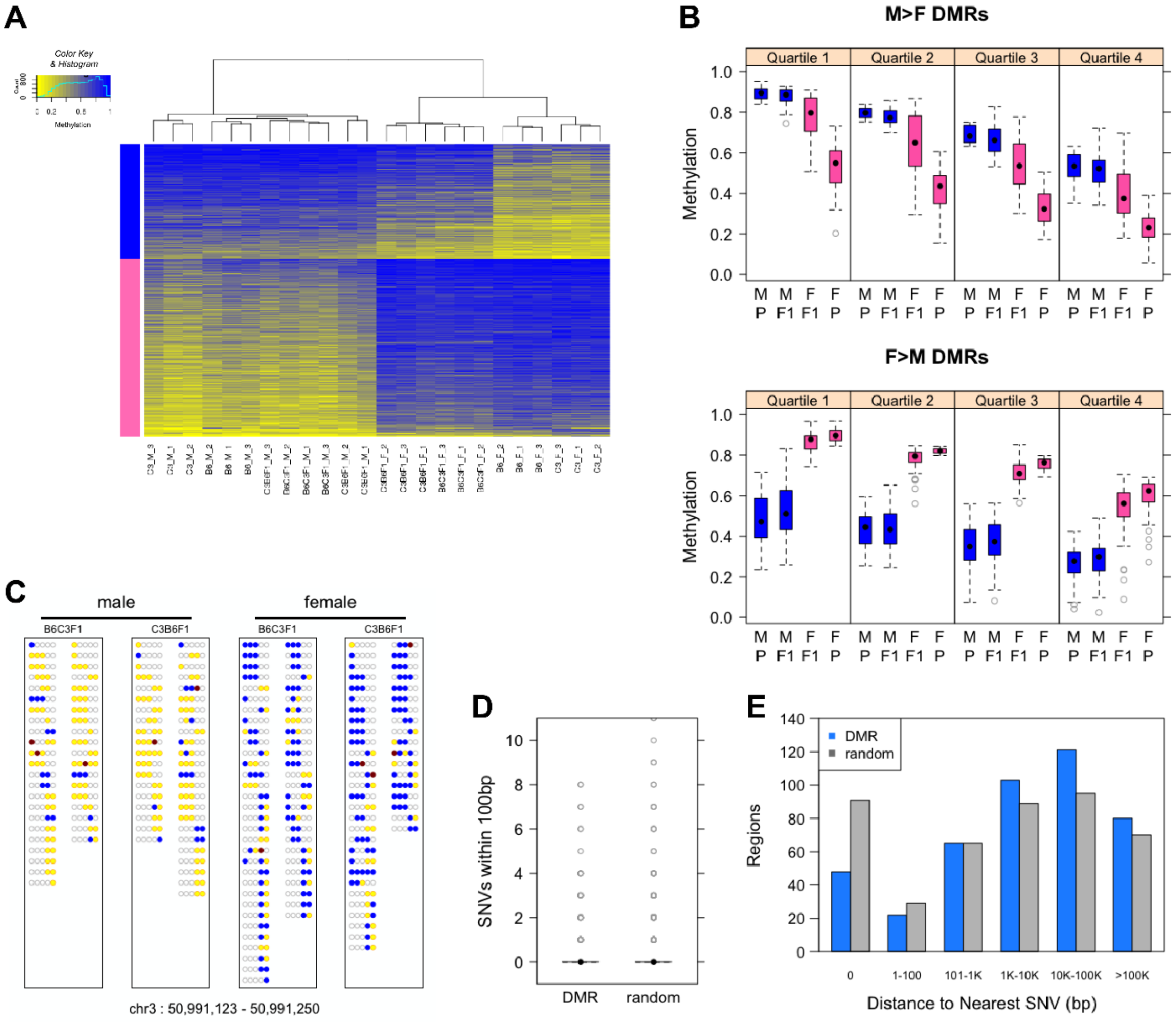

Supplementary Figure 9. Recapitulation of DNA methylation patterns at Metilene sex-DMRs in F1 progeny varies by DMR polarity.

A. Heatmap view of weighted methylation scores per Male-vs-Female Metilene DMR (N=439) per animal. Hierarchical clustering of animals peformed by R package 'amap' (hcluster with method=euclidean and link=average). DMRs were split by direction (M>F: blue bar, or F>M: pink bar) then sorted by average methylation score over all 24 animals.

B. Distribution of average weighted methylation scores, with DMRs split into quartiles according to decreasing methylation score of the hypermethylated parental sex. Animals are grouped according to both sex (M, F) and genotype group (P=parental B6 or C3, F1=offspring B6C3F1 or C3B6F1).

C. Methylation inheritance in F1 progeny at the read level for an exemplar Metilene DMR, organized as described for Fig3C.

D. Number of SNVs local to DMRs (N=439), compared to size-matched random genomic regions (N=439).

E. Distribution of distance to the nearest SNV for DMRs (N=439) or size-matched random genomic regions (N=439). Distance=0 indicates that at least one SNV is found within a region of a given type.

In the box-and-whisker plots, the box depicts the 25th to 75th percentiles, the black dot is the median, the whiskers extend to data points up to 1.5*IQR beyond the box, and open gray circles are data points outside the whisker range.

Grimm et al., Supplementary Figure 10

A M>F DMRs

Motif	Name	pvalue
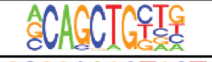	Tcf12(bHLH)	1e-7
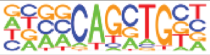	Ascl1(bHLH)	1e-6
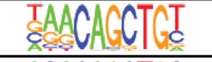	Myf5(bHLH)	1e-6
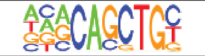	E2A(bHLH)	1e-5
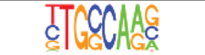	NF1-halfsite(CTF)	1e-5
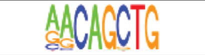	MyoG(bHLH)	1e-5
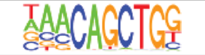	Tcf21(bHLH)	1e-5
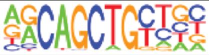	MyoD(bHLH)	1e-4
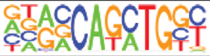	Atoh1(bHLH)	1e-4
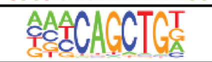	Ap4(bHLH)	1e-4
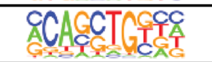	HEB(bHLH)	1e-4
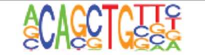	Ptf1a(bHLH)	1e-3
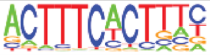	PRDM1(Zf)	1e-3
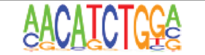	ZBTB18(Zf)	1e-3
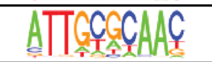	CEBP(bZIP)	1e-3
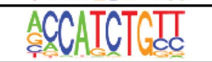	NeuroG2(bHLH)	1e-2
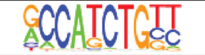	NeuroD1(bHLH)	1e-2
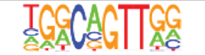	AMYB(HTH)	1e-2
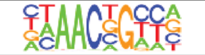	BMYB(HTH)	1e-2
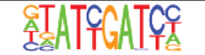	HNF6(Homeobox)	1e-2

B F>M DMRs

Motif	Name	pvalue
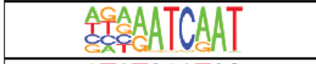	Cux2(Homeobox)	1e-15
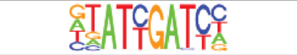	HNF6(Homeobox)	1e-15
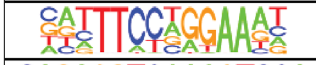	STAT1(Stat)	1e-4
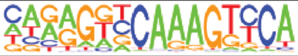	HNF4a(NR),DR1	1e-4
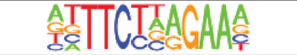	STAT5(Stat)	1e-4
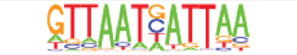	HNF1b(Homeobox)	1e-3
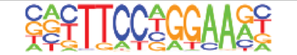	Stat3+il21(Stat)	1e-3
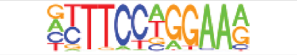	STAT4(Stat)	1e-3
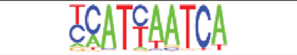	Pdx1(Homeobox)	1e-2
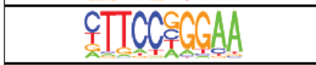	Stat3(Stat)	1e-2
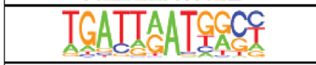	Hoxb4(Homeobox)	1e-2
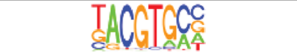	HIF-1a(bHLH)	1e-2
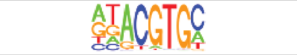	HIF-1b(HLH)	1e-2
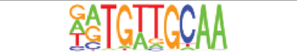	CEBP:AP1(bZIP)	1e-2
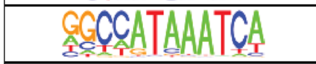	Hoxc9(Homeobox)	1e-2
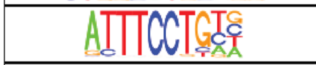	EWS:ERG-fusion (ETS)	1e-2
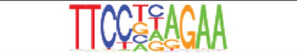	STAT6(Stat)	1e-2
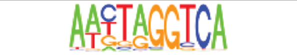	RORgt(NR)	1e-2
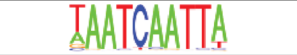	Pax7 (Paired,homeobox)	1e-2
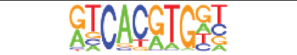	Usf2(bHLH)	1e-2

Supplementary Figure 10. Enriched motifs in Metilene sex-DMRs.

A. Top 20 enriched TF motifs in Male>Female Metilene DMRs (after expanding DMR size to a minimum of 401 bp) according to HOMER v4.9.1

B. Top 20 enriched TF motifs in Female>Male Metilene DMRs (after expanding DMR size to a minimum of 401 bp) according to HOMER v4.9.1

Supplementary Table 1

# Global methylation level calculated as "total methylated cytosine bases / total cytosine bases", summed over both strands.

# Only sites with validated context in both parental genomes are considered in these calculations.

	CpG	CHG	CHH
**animal**	76.58%	0.28%	0.27%
B6_M_1	76.98%	0.42%	0.41%
B6_M_2	76.69%	0.26%	0.25%
B6_M_3	75.24%	0.30%	0.29%
B6_F_1	75.70%	0.26%	0.25%
B6_F_2	75.03%	0.45%	0.44%
B6_F_3	77.11%	0.39%	0.40%
C3_M_1	77.31%	0.34%	0.36%
C3_M_2	76.97%	0.54%	0.54%
C3_M_3	76.22%	0.35%	0.36%
C3_F_1	77.05%	0.55%	0.54%
C3_F_2	76.90%	0.31%	0.32%
C3_F_3	76.89%	0.39%	0.39%
B6C3F1_M_1	76.94%	0.55%	0.53%
B6C3F1_M_2	76.28%	0.43%	0.44%
B6C3F1_M_3	76.39%	0.39%	0.39%
B6C3F1_F_1	76.42%	0.48%	0.47%
B6C3F1_F_2	75.92%	0.48%	0.47%
B6C3F1_F_3	76.42%	0.38%	0.37%
C3B6F1_M_1	77.03%	0.47%	0.46%
C3B6F1_M_2	77.55%	0.28%	0.28%
C3B6F1_M_3	76.26%	0.39%	0.38%
C3B6F1_F_1	76.67%	0.46%	0.45%
C3B6F1_F_2	76.84%	0.28%	0.28%
C3B6F1_F_3			

	CpG	CHG	CHH
**group averages**	76.04%	0.33%	0.32%
B6	76.93%	0.41%	0.42%
C3	76.47%	0.45%	0.45%
B6C3F1	76.79%	0.38%	0.37%
C3B6F1			

Supplementary Table 2

# Comparison of percent methylation per CpG site (minimum read depth 1, 5, or 10).

Animal	*Library Comparison*	*Minimum Required Depth = 1*		*Minimum Required Depth = 5*		*Minimum Required Depth = 10*	
		Qualifying CpG Sites	*PearsonR*	*Qualifying CpG Sites*	*PearsonR*	*Qualifying CpG Sites*	*PearsonR*
B6_F_1	L1 vs L2	17,431,041	0.7141	5,252,433	0.7300	191,193	0.7687
	L1 vs L3	18,661,769	0.8648	15,937,090	0.8777	7,569,263	0.8704
	L2 vs L3	17,477,591	0.7563	5,488,372	0.7752	226,731	0.8006
B6_F_2	L1 vs L2	18,689,317	0.8798	17,035,256	0.8833	11,115,326	0.8725
B6_F_3	L1 vs L2	18,738,857	0.8980	17,831,611	0.9100	14,477,624	0.9082
B6_M_1	L1 vs L2	17,133,992	0.7133	3,927,961	0.7403	87,118	0.7960
	L1 vs L3	18,692,567	0.8816	16,994,539	0.8908	10,824,468	0.8829
	L2 vs L3	17,156,983	0.7407	3,996,612	0.7714	94,942	0.8157
B6_M_2	L1 vs L2	18,699,805	0.8945	17,417,134	0.8985	13,096,465	0.8847
B6_M_3	L1 vs L2	18,546,540	0.8512	15,336,917	0.8417	6,440,645	0.8348
C3_F_1	L1 vs L2	17,001,232	0.7194	5,437,928	0.7429	263,895	0.7801
	L1 vs L3	18,421,843	0.8594	15,490,939	0.8853	7,965,713	0.8831
	L2 vs L3	17,052,580	0.7558	5,643,253	0.7807	317,465	0.8026
C3_F_2	L1 vs L2	18,454,773	0.8753	16,358,300	0.8861	11,383,229	0.8734
C3_F_3	L1 vs L2	18,216,575	0.8333	13,981,205	0.8349	5,128,227	0.8313
C3_M_1	L1 vs L2	16,425,637	0.6806	3,225,685	0.7209	62,842	0.7992
	L1 vs L3	18,292,105	0.8391	13,097,335	0.8664	3,965,654	0.8606
	L2 vs L3	16,575,058	0.7407	3,657,899	0.7845	102,075	0.8292
C3_M_2	L1 vs L2	18,474,773	0.8764	16,287,661	0.8916	10,582,098	0.8828
C3_M_3	L1 vs L2	18,538,148	0.8902	16,974,650	0.9112	12,876,498	0.9073
B6C3F1_F_1	L1 vs L2	17,420,509	0.7334	5,312,553	0.7563	183,766	0.7985
	L1 vs L3	18,677,782	0.8820	16,801,992	0.9007	10,599,854	0.9059
	L2 vs L3	17,455,894	0.7584	5,477,415	0.7828	224,764	0.8129
B6C3F1_F_2	L1 vs L2	17,797,970	0.7702	6,503,498	0.7967	284,209	0.8283
	L1 vs L3	17,791,709	0.7665	6,460,042	0.7925	271,533	0.8255
	L2 vs L3	18,705,503	0.8905	17,315,412	0.9015	12,776,951	0.8954
B6C3F1_F_3	L1 vs L2	18,711,772	0.8964	17,535,985	0.9110	13,346,645	0.9124
B6C3F1_M_1	L1 vs L2	13,184,262	0.6119	386,907	0.7032	3,902	0.9250
	L1 vs L3	18,629,127	0.8611	15,493,510	0.8751	6,674,978	0.8674
	L2 vs L3	13,214,224	0.6415	403,530	0.7363	4,010	0.9258
B6C3F1_M_2	L1 vs L2	18,665,313	0.8749	16,470,713	0.8754	9,091,070	0.8650
B6C3F1_M_3	L1 vs L2	18,619,698	0.8657	15,219,481	0.8750	5,925,464	0.8625
C3B6F1_F_1	L1 vs L2	15,001,853	0.6317	1,011,083	0.6743	18,753	0.7671
	L1 vs L3	18,608,663	0.8574	15,416,285	0.8790	7,026,231	0.8779
	L2 vs L3	15,077,087	0.6667	1,131,388	0.7115	38,175	0.7209
C3B6F1_F_2	L1 vs L2	18,692,004	0.8882	17,140,387	0.8904	12,395,436	0.8789
C3B6F1_F_3	L1 vs L2	18,532,308	0.8509	15,281,380	0.8413	6,974,601	0.8318
C3B6F1_M_1	L1 vs L2	17,235,341	0.7274	4,454,765	0.7587	118,593	0.7980
	L1 vs L3	18,685,764	0.8817	16,901,932	0.8980	11,223,597	0.8983
	L2 vs L3	17,260,478	0.7495	4,530,737	0.7824	128,067	0.8153
C3B6F1_M_2	L1 vs L2	18,652,380	0.8840	16,948,466	0.8844	11,846,790	0.8700
C3B6F1_M_3	L1 vs L2	18,470,559	0.8416	14,661,549	0.8281	5,681,844	0.8202
MIN			*0.6119*		*0.6743*		*0.7209*
MAX			*0.8980*		*0.9112*		*0.9258*
MEDIAN			*0.8462*		*0.8415*		*0.8615*
