# Supplementary material for: DNA methylation in mice is influenced by genetics as well as sex and life experience
Source: Nat Commun. 2019 Jan 18;10:305. doi: 10.1038/s41467-018-08067-z (PMC6338756; doi:10.1038/s41467-018-08067-z)
Supplement: Supplementary file 3 — Description of Additional Supplementary Files [file 41467_2018_8067_MOESM3_ESM.pdf]

Description of Additional Supplementary Files

File Name: Supplementary Data 1
Description: DNA-seq Tab` Sequencing counts and quality checks for DNA-seq data, including raw read counts, filtered read counts (require minimum average base quality score of 20), mapping efficiency (unmapped, low quality, or high quality hits), duplication rate, and approximate average depth per sequencing run and per animal. BS-seq Tab` Sequencing counts and quality checks for BS-seq data, including raw read counts per library, filtered read counts (require minimum average base quality score of 20), bisulfite conversion rate per library, mapping efficiency (unmapped, ambiguous hits, or mapped uniquely to mm9 or lambda), duplication rate, and approximate average depth per animal for both the mm9 genome and lambda. Approximate average depth is calculated after clipping read cycles with unacceptable positional methylation bias based on M-Bias plot and after clipping overlapping mapped ends from the same read pair.

File Name: Supplementary Data 2
Description: Lists of DMRs from B6-vs-C3 comparison via DSS or Metilene. Comparisons were performed using animals from only parental genotypes (i.e. B6 or C3, N=6 animals per query group). Each DMR is annotated according to polarity type (B6>C3 or C3>B6), size, number of validated CpG sites, weighted methylation scores per query group, mean depth per query group, distance to nearest TSS (based on RefSeq as of Feb 8 2016), and identity of nearest gene (reported as Entrez gene symbol and Entrez gene ID).

File Name: Supplementary Data 3
Description: Lists of DMRs from male-vs-female comparison via DSS or Metilene. Comparisons were performed using animals from both parental genotypes (i.e. B6 and C3, N=6 animals per query group), only one parental genotypes (i.e. B6 or C3, N=3 animals per query group), or the F1 genotypes (i.e. B6C3F1 and C3B6F1, N=6 animals per query group). Each DMR is annotated according to polarity type (M>F or F>M), size, number of validated CpG sites, weighted methylation scores per query group, mean depth per query group, distance to nearest TSS (based on RefSeq as of Feb 8 2016), and identity of nearest gene (reported as Entrez gene symbol and Entrez gene ID).

File Name: Supplementary Data 4
Description: Lists of DMRs from mothers-vs-virgins comparison via DSS or Metilene. The comparison was for female animals from both parental genotypes (i.e. B6 and C3, N=6 animals) versus female animals from both F1 genotypes (B6C3F1 and C3B6F1, N=6 animals). Each DMR is annotated according to polarity type (vrgs>moms or moms>vrgs), size, number of validated CpG sites, weighted methylation scores per query group, mean depth per query group, distance to nearest TSS (based on RefSeq as of Feb 8 2016), and identity of nearest gene (reported as Entrez gene symbol and Entrez gene ID).
